# CTCA in children with severe heterozygous familial hypercholesterolaemia: Screening for subclinical atherosclerosis

**DOI:** 10.1016/j.athplu.2023.12.002

**Published:** 2023-12-13

**Authors:** M. Doortje Reijman, Sibbeliene E. van den Bosch, D. Meeike Kusters, Willemijn E. Corpeleijn, Barbara A. Hutten, Irene M. Kuipers, R. Nils Planken, Albert Wiegman

**Affiliations:** a Amsterdam UMC Location University of Amsterdam, Department of Pediatrics, Amsterdam Cardiovascular Sciences, Amsterdam Gastroenterology Endocrinology Metabolism, Meibergdreef 9, Amsterdam, the Netherlands; b Amsterdam UMC Location University of Amsterdam, Department of Epidemiology and Data Science, Amsterdam Cardiovascular Sciences, Meibergdreef 9, Amsterdam, the Netherlands; c Amsterdam UMC Location University of Amsterdam, Department of Radiology and Nuclear Medicine, Amsterdam Cardiovascular Sciences, Meibergdreef 9, Amsterdam, the Netherlands

**Keywords:** Familial hypercholesterolemia, Severe HeFH, LDL-C, Children, Atherosclerosis, CTCA, Imaging

## Abstract

Familial hypercholesterolemia (FH) is one of the most common genetically inherited disorders in the world. Children with severe heterozygous FH (HeFH), i.e. untreated low-density lipoprotein cholesterol (LDL-C) levels above the 90th percentile for age and sex among FH mutation carriers, can have LDL-C levels that overlap levels of children with homozygous FH (HoFH), but treatment regimen and cardiovascular follow-up to prevent cardiovascular disease are less intensive in children with severe HeFH. In children with HoFH, subclinical atherosclerosis can already be present using computed tomography coronary angiography (CTCA). The question remains whether this is also the case in children with severe HeFH who have a high exposure to elevated LDL-C levels from birth onwards as well. We calculated the cumulative LDL-C exposure (CE_total_ [mmol]) in four children with severe HeFH and performed computed tomography coronary angiography (CTCA). These children, aged 13, 14, 15 and 18 years, had CE_total_ of 71.3, 97.8, 103.6 and 136.1 mmol, respectively. None of them showed abnormalities on cardiovascular imaging, despite high LDL-C exposure. The results of this study, do not give us an indication to recommend performing CTCA routinely in children with severe HeFH.

## Highlights

• Children with severe HeFH have untreated LDL-C levels >90th percentile.
• LDL-C levels in children with severe HeFH can overlap with those in HoFH children.
• CTCA is used as routinely screening for atherosclerosis in HoFH children.
• Four children with severe HeFH underwent CTCA and none of these had abnormalities.
• The results of this study do not support routine CTCA in children with severe HeFH.

## 1 Introduction

Familial hypercholesterolemia (FH) is an inherited disorder of lipoprotein metabolism. It is caused by genetic variants in key proteins involved in LDL-C metabolism and characterized by severely elevated LDL-C levels [1]. Whereas heterozygous familial hypercholesterolemia (HeFH) is common, homozygous familial hypercholesterolemia (HoFH) is rare but much more lethal. This is due to genetic variants in two alleles and therefore extremely increased LDL-C levels. Left untreated, children at age 4 and 5 years have died due to myocardial infarction [2].Therefore, children with HoFH require intensive lipid-lowering therapy (LLT) from diagnosis onwards and regular screening for subclinical atherosclerosis with echocardiography and computed tomography coronary angiography (CTCA) is recommended [3]. More LLT, including proprotein convertase subtilisin/kexin type 9 inhibitors (PCSK9-i), angiopoietin-like 3 inhibitors (ANGTPL3-i) and lipoprotein apheresis are available for children with HoFH and at a younger age, compared to HeFH. Because of the normally less severe phenotype of HeFH, LLT is only started from the age of 8 years and there are no recommendations regarding routinely screening for subclinical atherosclerosis. However, severity of phenotype in both HoFH and HeFH can vary in a broad range, and LDL-C levels of some HeFH children can overlap with LDL-C levels of children with HoFH [4]. It is known that higher LDL-C levels are associated with a dose-dependent increased risk of cardiovascular disease (CVD). Besseling et al. (2014) proposed a definition for patients with *severe* HeFH, with a significant higher risk of CVD. Severe FH was defined as LDL-C levels above the 90th percentile for age and sex among FH mutation carriers, making it also applicable for children [5]. In children with HoFH calcified and non-calcified plaques can already be detected by CTCA, despite effective LLT with lipoprotein apheresis: a therapy not administered in children with HeFH [6,7]. Considering the overlap in LDL-C levels between HoFH and severe HeFH, the question is whether atherosclerotic manifestations are already exhibited in children with severe HeFH. This could implicate a treatment regime similar to that of HoFH children. Therefore, we study the cumulative LDL-C exposure and the results of CTCA of four children with severe HeFH in the light of LLT and screening for subclinical atherosclerosis in children with severe HeFH.

## 2 Methods

### 2.1 Study population and design

Our study population consisted of children who were under treatment in our outpatient clinic (Amsterdam University Medical Center – Location AMC) specialized in treatment of children with FH. We included children with untreated LDL-C levels above the 90th percentile for age and sex in children with HeFH, fulfilling the criteria for severe HeFH and underwent CTCA of the heart with contrast to screen for subclinical atherosclerosis [5]. This study complies with the Declaration of Helsinki. As this retrospective study was not subject to the Medical Research Involving Human Subjects Act, approval from an ethics committee in the Netherlands was not required.

### 2.2 Cumulative LDL-C exposure

For each child, we calculated the cumulative LDL-C exposure ([CE_total_] mmol). This CE_total_ consists of LDL-C exposure (mmol x years) before initiating LLT, during treatment with oral LLT, and during treatment with PCSK9-i, if applicable.

### 2.3 Imaging

A third-generation dual source CT-scanner (Siemens Somatom Force, Germany) was used to evaluate subclinical atherosclerosis. A prospective ECG-triggered high-pitch spiral scan sequence was applied for children in sinus rhythm and a heartrate below 70 beats per minute and a prospective ECG-triggered adaptive sequential scan sequence was applied in children with irregular heart rhythm or heartrates >70 beats per minute. Intravenous iodine-based contrast medium (Ultravist 300 mg/ml, Bayer Healthcare Pharmaceuticals, Berlin, Germany) was administered via venous access at the antecubital fossa with an injection speed adjusted to body weight and tube voltage [8]. Scans were analyzed by one experienced cardiovascular radiologist using dedicated coronary post-processing software (SyngoVia, Siemens). Coronary anatomy, vessel wall irregularities by calcified plaque, mixed plaque or non-calcified plaque and luminal stenosis were assessed.

### 2.4 Statistical analysis

Descriptive statistics were used to summarize demographic and clinical characteristics of children, as well as the outcomes obtained from CTCA.

## 3 Results

### 3.1 Description of study population

Four children with severe HeFH, aged 13, 14, 15 and 18 years underwent CTCA to screen for subclinical atherosclerosis. All four patients recently entered an RCT on inclisiran, a small interfering RNA molecule directed against PCSK9 (PCSK9-i) [9]. Two of the children (patient 2 and 3) participated before in an RCT on alirocumab, a monoclonal antibody directed against PCSK9 (PCSK9-i) [10]. Demographic and clinical characteristics are summarized in Table 1. All were genetically diagnosed with HeFH based on receptor-negative variants in *LDLR*. Next-generation sequencing of 27 genes causally associated with genetic dyslipidemias, including *APOB* and *PCSK9* showed no other pathogenic variants. Family history showed the first premature CVD in first- or second-degree family members, aged 38–42 years (Table 1).

**Table 1:** Demographic and clinical characteristics of four children with heterozygous familial hypercholesterolemia.

			Patient 1	Patient 2	Patient 3	Patient 4
Age (years)	At diagnosis		7	5	6	14
	At initiation of oral LLT		8	7	8	15
	At start of PCSK9-i RCT		13^a^	8^b^	9^b^	15^a^
	At CT scan		13	14	15	18
Sex			Male	Male	Female	Female
Premature CVD in family	Present		Yes	Yes	Yes	Yes
	Degree		1st degree	2nd degree	2nd degree	2nd degree
	Age of CVD (years)		42	38	38	38
Clinical signs	Xanthoma's/arcus corneae		No	No	No	No
Genetic variant	Gene		*LDLR*	*LDLR*	*LDLR*	*LDLR*
	Mutation		c. (190 + 1_191-1)_ (1186 + 1_1187-1) dup p.?	c.1359-1G > A p.?	c.1359-1G > A p.?	c.313+1G > A p.?
	Type		Negative	Negative	Negative	Negative
LLT at CT			Rosuvastatin 10 mg	Rosuvastatin 10 mg	Rosuvastatin 10 mg	Pravastatin 20 mg Ezetemib 10 mg
Lipoprotein(a) (mg/dL)			18.0	8.3	38.0	6.4
LDL-C (mmol/L)	Untreated		6.6	8.4	8.0	7.9
	On oral LLT		3.7	6.4	5.8	6.8
	At CT scan		3.7	3.5^c^	5.3^c^	5.4^c^
	Cumulative LDL-C exposure		71.3	86.2	101.6	134.7
Cardiovascular Imaging	CT-CAC	Calcified coronary plaques	N/A	No	No	No
	CTCA	Coronary arteries Signs of CAD	Normal No	Normal No	Normal No	Normal No

CAD, coronary artery disease; CT−CAC, computed tomography coronary artery calcium score; CCTA, coronary computed tomography; CVD, cardiovascular disease; <LDLR, LDL-receptor; LDL−C, low-density lipoprotein cholesterol; LLT, lipid-lowering therapy; mmol/L, millimole per litre; N.A., not applicable; PCSK9−i, proprotein convertase subtilisin/kexin type 9 inhibitor; RCT, randomized controlled trial.

a Inclisiran.

b Alirocumab.

c during RCT with PCSK9-i.

### 3.2 Cumulative LDL-C exposure and cardiovascular imaging

At the moment of CT scan, these children had LDL-C levels above the treatment target of 3.4 mmol/L, ranging from 3.5 to 5.4 mmol/L. The cumulative LDL-C exposure at time of imaging (CE_total_) varied from 71.3 to 134.7 mmol. None of the children had elevated lipoprotein(a) levels exceeding the 50 mg/dL cutoff. All four children underwent cardiac CT. Three children (subject 2, 3 and 4) underwent a CT-coronary artery calcium score (CT-CAC) and CTCA. One child (subject 1) underwent only CTCA. The cumulative radiation dose varied from 0.3 to 2.6 (median 0.5) mSv. None of these four children showed abnormalities on cardiovascular imaging (Table 1).

## 4 Discussion

We describe four children with severe HeFH who had a CTCA for cardiovascular follow-up. Despite the high CE_total_, the scans of these children did not show any signs of atherosclerosis. In adults with HeFH, treated with long-term statin treatment, subclinical coronary atherosclerosis was present in the majority (85 %) (mean age 52 ± 7 years) [11]. To the best of our knowledge, this is the first study describing screening for subclinical coronary atherosclerosis in children (13–18 years of age) with severe HeFH. The results of this study, did not give us an indication to recommend performing screening for subclinical atherosclerosis in all children with severe HeFH. If there are indications (i.e. cardiac symptoms or other CVD risk factors than FH, etcetera) for an individual to screen for signs of subclinical atherosclerosis we do recommend to use CTCA if available, because of the low radiation dose and the high sensitivity for subclinical atherosclerosis. The median cumulative radiation dose in the four described children was 0.5 mSv, comparable with ∼3 chest X-rays. However, the maximum dose necessary was 2.6 mSv, due to factors as a high and irregular heartrate. If the likelihood of detecting signs of subclinical atherosclerosis is low, as indicated by the results in our children with severe HeFH, it is not recommended to subject children to these levels of radiation.

An LDL-C burden of 160 mmol is sufficient to develop coronary heart disease [12]. Literature indicated that healthy adults reach this burden at the age of 55, while children with HoFH already reach this level at the age of 12.5 or even earlier. This is in line with a study describing CTCA results of six children with HoFH. Three children described in that study had a higher CE_total_ (range 157.4 mmol–208.9 mmol) and showed abnormalities on the CTCA, whereas children with a lower CE_total_ (41.7 and 64.3 mmol) showed no abnormalities. One patient with a CE_total_ of 144.0 mmol also exhibited abnormalities; however, she had a known history of untreated LDL-C levels above 20 mmol/L for several years what could have contributed to these findings on CTCA. Considering the four children with severe HeFH described in this study have a CE_total_ in the range of 71.3–134.7 mmol at the age of 13–18 years, this indicates that these children with severe HeFH do not have a similar cumulative LDL-C exposure as children with HoFH. This could explain the absence of subclinical atherosclerosis in these patients with severe HeFH. The question remains if children with long-time untreated high LDL-C levels comparable with HoFH patients, would show signs of subclinical atherosclerosis on imaging.

There are some aspects of our study that merit discussion. Because of the small sample size, we cannot exclude that patients with severe HeFH do not develop subclinical atherosclerosis in childhood, specifically children with even higher cumulative LDL-C exposure. As compared before with HoFH children, LDL-C exposure in our severe HeFH children may not be sufficient to develop cardiovascular abnormalities. As all four children recently started to participate in a randomized trial, we were blinded for treatment (inclisiran or placebo 2:1) at time of imaging [9]. If children received inclisiran before the CTCA was performed, there could be a chance that, due to the reduction of LDL-C levels, the CTCA would have improved [13]. However, we expect this effect to be minimal, because children received one single dose of the study drug less than 3 months before imaging was performed.

To investigate why these children with severe HeFH in our study did not show signs of subclinical atherosclerosis, future research should include CTCA results of children with HeFH with a cumulative LDL-C exposure above 160 mmol, to confirm absence of cardiovascular abnormalities. Those results would be helpful for deciding if guidelines and availability of LLT should be specified for HeFH and HoFH patients, or should be specified on the cumulative LDL-C exposure of an individual.

## 5 Conclusion

Our study shows that four children with severe HeFH did not have any signs of subclinical atherosclerosis on CTCA, despite high LDL-C exposure up to 134.7 mmol. The results of this study, do not give us an indication to recommend performing CTCA routinely in children with severe HeFH.

## Author’s contributions

SEvdB and MDR performed the conceptualization and design of the study. SEvdB and MDR wrote the manuscript. SEvdB, MDR, DMK, RNP, AW performed the interpretation of the data. DMK, RNP, WEC, BAH, IMK and AW revised the manuscript. All authors approved the final version of the manuscript

## Declaration of competing interest

The authors declare the following financial interests/personal relationships which may be considered as potential competing interests:

AW reports research support from pharmaceutical trials of lipid modification agents from Amgen, Regeneron, Novartis, 10.13039/100010137Sanofi, 10.13039/501100022336Esperion, 10.13039/501100022030Silence Therapeutics and Ultragenyx, and is a member of the safety board at Amryt. BAH and AW received a research grant from Silence Therapeutics. SEvdB, MDR, DMK, IMK, RNP and WEC have no conflicts of interest.
